# Modelling of salt intake reduction by incorporation of umami substances into Japanese foods: a cross-sectional study

**DOI:** 10.1186/s12889-023-15322-6

**Published:** 2023-03-19

**Authors:** Shiori Tanaka, Daisuke Yoneoka, Aya Ishizuka, Megumi Adachi, Hitomi Hayabuchi, Toshihide Nishimura, Yukari Takemi, Hisayuki Uneyama, Haruyo Nakamura, Kaung Suu Lwin, Kenji Shibuya, Shuhei Nomura

**Affiliations:** 1 Department of Global Health Policy, Graduate School of Medicine, The University of Tokyo, Tokyo, Japan; 2 Epidemiology and Prevention Group, Institute for Cancer Control, National Cancer Center, Tokyo, Japan; 3 Center for Surveillance, Immunization, and Epidemiologic Research, National Institute of Infectious Diseases, Tokyo, Japan; 4 Tokyo Foundation for Policy Research, Tokyo, Japan; 5 Graduate School of Health and Environmental Sciences, Fukuoka Women’s University, Fukuoka, Japan; 6 Faculty of Nutrition, Kagawa Nutrition University, Saitama, Japan; 7 Ajinomoto Co., Inc., Tokyo, Japan; 8 Department of Health Policy and Management, School of Medicine, Keio University, 35 Shinanomachi, Shinjuku-ku, Tokyo, 160-8582 Japan

**Keywords:** Sodium, Salt, Umami, Glutamate, Inosinate, Guanylate, Dietary goal, Japan

## Abstract

**Background:**

Evidence has demonstrated that excess sodium intake is associated with development of several non-communicable diseases. The main source of sodium is salt. Therefore, reducing salt intake in foods is an important global public health effort to achieve sodium reduction and improve health. This study aimed to model salt intake reduction with 'umami' substances among Japanese adults. The umami substances considered in this study include glutamate or monosodium glutamates (MSG), calcium diglutamate (CDG), inosinate, and guanylate.

**Methods:**

A total of 21,805 participants aged 57.8 years on average from the National Health and Nutrition Survey was used in the analysis. First, we employed a multivariable linear regression approach with overall salt intake (g/day) as a dependent variable, adjusting for food items and other covariates to estimate the contribution of salt intake from each food item that was selected through an extensive literature review. Assuming the participants already consume low-sodium products, we considered three scenarios in which salt intake could be reduced with the additional umami substances up to 30%, 60% and 100%. We estimated the total amount of population-level salt reduction for each scenario by age and gender. Under the 100% scenario, the Japan’s achievement rates against the national and global salt intake reduction goals were also calculated.

**Results:**

Without compromising the taste, the 100% or universal incorporation of umami substances into food items reduced the salt intake of Japanese adults by 12.8–22.3% at the population-level average, which is equivalent to 1.27–2.22 g of salt reduction. The universal incorporation of umami substances into food items changed daily mean salt intake of the total population from 9.95 g to 7.73 g: 10.83 g to 8.40 g for men and 9.21 g to 7.17 g for women, respectively. This study suggested that approximately 60% of Japanese adults could achieve the national dietary goal of 8 g/day, while only 7.6% would meet the global recommendation of 5.0 g/day.

**Conclusions:**

Our study provides essential information on the potential salt reduction with umami substances. The universal incorporation of umami substances into food items would enable the Japanese to achieve the national dietary goal. However, the reduced salt intake level still falls short of the global dietary recommendation.

## Main text

### Background

The latest Global Burden of Disease Study 2019 (GBD) highlighted that the global prevalence of non-communicable diseases (NCDs) and inadequate public health efforts to control risk factors may have spurred the pandemic of coronavirus disease 2019 (COVID-19) [1]. In 2013, the World Health Organization (WHO) developed the NCDs Global Monitoring Framework, in which nine NCDs prevention targets were set [2]. Of the nine targets, the only target specifically related to nutrients is a 30% relative reduction in mean population intake of salt/sodium between 2011 and 2025 [2]. Since then, many campaigns aiming at reducing salt, the main source of sodium, have been initiated around the world [3], and the global salt reduction movement has been accelerated [4–6]. However, no country has yet to achieve the 30% reduction goal [7]. In the GBD 2019, high salt intake was listed as one of the top dietary risks contributing to the global burden of disease [8] highlighting the need for an urgent approach.

Japan is one of the countries that are globally recognized for prolonged longevity [9]. However, a high salt intake is a major dietary risk factor for both mortality and morbidity of its population [8, 10]. Japan's nationwide population-based campaign for salt reduction started in 1960s and successfully reduced the population's salt intake and mortality resulting from stroke over time [11]. According to the National Nutrition Survey (NNS), which was renamed to the National Health and Nutrition Survey (NHNS) in 2013, the daily salt intake has steadily decreased from 14.5 g in 1973 to 9.5 g in 2017 [12]. However, the Japanese generally consume more salt than people in other countries [13]. For instance, the population average sodium intake in 2010 was 4.89 g/day (12.23 g/day of salt intake) in Japan, whereas those in the United Kingdom (UK) and the United States (US) were 3.61 g/day (9.03 g/day of salt intake) and 3.60 g/day (9.00 g/day of salt intake), respectively [13]. The government aims to reduce the daily salt intake of Japanese adults to 8 g by 2023 in their 10-year national health promotion plan, titled the Second Term of National Health Promotion Movement in the Twenty-First Century, also known as "Health Japan 21 (the second term)" [14]. Another dietary guideline is called the Dietary Reference Intakes for Japanese (DRIs), which proposes reference values for the intake of energy and nutrients to prevent lifestyle-related diseases and extend healthy life expectancy [15]. The DRIs recommend daily salt intake of 7.5 g/day for men and 6.5 g/day for women. However, the average salt intake among Japanese adults remains higher than the recommendations made by both guidelines. The targets set for the Japanese is unlikely to be attained if current trends persist [16, 17].

Sodium replacement in foods is one of the most widely used approaches to reduce salt intake. The technical challenge is to ensure that the sodium alternative is palatable and safe to eat [18]. Umami is a common and familiar taste in Japanese cuisine, and perhaps globally better known as the fifth flavour, in addition to the classic four tastes: saltiness, sweetness, bitterness, and sourness, discovered by the Japanese scientist in 1908 [19]. Umami substances, including glutamate or monosodium glutamates (MSG), calcium diglutamate (CDG), inosinate and guanylate, have been proposed as enhancers of savory taste when combined with sodium chloride (NaCl) [20–22]. A large number of studies have suggested the potential use of umami substances as a healthy and natural solution for salt intake reduction [23–25]. In recent years, academic institutions, such as the Institute of Medicine in the United States, have identified umami substances as candidates for practical salt intake reduction alternatives [18]. Wallace et al. (2019) estimated that incorporating MSG into a savoury seasoning of processed foods in the United States could reduce salt intake of the population by at least 3 to 8% [26]. However, given the fact that the source of salt intake is highly dependent on the dietary habits and the cooking processes in each country [27], the effectiveness of the umami substances for reducing salt intake at the population level in the context of other cultures is not well known. Therefore, our study aims to investigate the effects of umami substances on the daily salt (NaCl) intake reduction among Japanese adults using the NHNS data.

## Methods

### Study design and participants

We conducted a cross-sectional study using the de-identified national data from the 2016 NHNS. The NHNS is a nationally representative household survey conducted annually by the Japanese Ministry of Health, Labour and Welfare (MHLW) to collect data on the population's dietary habits, nutrition intake and lifestyle [28]. Residents above the age of one were selected from the census enumeration areas using a stratified single-stage cluster sample design. The 2016 survey, the latest large-scale survey data available from the NHNS at the time of the study, is comprised of 24,187 households randomly selected from 475 districts. The response rate of the survey was 44.4%. The NHNS consists of three parts: 1) physical examination, 2) an in-person survey and weighted single-day dietary record of households, and 3) a self-reported lifestyle questionnaire. Details of the survey design and the procedures are available elsewhere [16, 28]. In the present analysis, we included persons aged 20 years or older as Health Japan 21 requires the age group to complete the dietary intake data. We further excluded participants who reported daily consumption of less than 1.5 g of salt, the minimum physiological requirement for survival, assuming that the data may not reflect their diet accurately or may be measurement error.

This study was performed under the Declaration of Helsinki and approved by the Research Ethics Committee of the Graduate School of Medicine, the University of Tokyo (authorization number 11964). The ethical committee waived the need for informed consent because this study conducted a secondary analysis using anonymized data routinely collected by the MHLW.

### Dietary assessment

The dietary intake survey was conducted on a single designated day for household representatives, who were usually responsible for food preparation. Trained interviewers, mainly registered dietitians, instructed household representatives on how to measure the food and beverages consumed by members of the household and checked their compliance with the survey. The proportion of shared dishes, food waste, and foods eaten out were recorded by the household representatives. The nutrient intake and food consumption were estimated by experts using the dietary record and the corresponding food composition list of the Japanese Standard Tables of Food Composition (7^th^ revised edition) [29]. In addition, food intake (g/day) and overall sodium intake (mg/d) were recorded. Salt intake (g) was defined as sodium (mg) × 2.54/1,000. NHNS did not measure urine sodium.

### Salt intake modelling

We have conducted an extensive review of the scientific literatures, and found that umami substances, such as glutamate or MSG, CDG, inosinate, and guanylate, have been used to reduce salt in various mainstream products. Table 1 shows the percentage reduction of salt intake in each food item estimated by previous studies using one or more umami substances. The food items listed in Table 1 were then matched with the predetermined 13 NHNS food groups, and assumed the salt reduction rate for each NHNS food group to be used in our analyses, in consultation with food and nutrition experts (co-authors). In addition, we assumed that the study participants already consume some low-sodium food items containing umami substances in their diet. Therefore, the market share of low-sodium food products was used as a proxy indicator of the baseline consumption of low-sodium foods [30]. This market share was estimated from data of the total sales and the sales of low-sodium foods acquired from the surveys conducted in 2017 by Fuji Keizai Management Co., Ltd. (a Japanese market research company). Low-sodium food products were defined as products labelled with “reduced salt,” “salt cut,” “salt off,” or “no salt” on the package. The market share of low-sodium food products for each food group was also summarized in Table 1. We considered three scenarios in which consumers could potentially reduce their salt intake with the additional umami substances from the baseline to 30% (30-percent scenario), 60% (60-percent scenario) and 100% (100-percent scenario or universal incorporation scenario).

**Table 1:** Percentage reduction of salt intake in food items by incorporation of umami substance

References	Food items	Salt alternatives	Salt reduction rate (%)	NHNS food group	Assumed salt reduction rate in the study (%)	Assumed market share of low-sodium products (%)
JPA 01–304860 [31]	Salt composition	MSG, inosinate	22–43	Seasoning salt	22–43	9.2
JPA 58–198269 [32]	Salt composition	MSG, inosinate, guanylate	30			
JPA 2006–141226 [33]	Liquid seasoning	MSG	40–49	Soy sauce	40–61	28.7
Ishida 2011 [34]	Soy sauce	MSG, inosinate, guanylate	60			
JPA 09–275930 [35]	Soy sauce	MSG, inosinate, guanylate	61			
Ishida 2011 [34]	Miso paste	MSG, inosinate, guanylate	15	Miso paste	15–35	12.4
JPA5523618 [36]	Low-salt bean miso	Glutamine acid	35			
Chi 1992 [37]	Chicken broth	MSG	11	Other seasonings	11–40	4.1
Manabe 2008 [38]	Japanese clear soup	Dried bonito (Rich in inosinate)	15			
Goh 2011 [39]	Japanese clear soup	Soy sauce (Rich in glutamates)	15			
Huynh 2016 [40]	Tomato sauce	Fish sauce (Rich in glutamates)	16			
Kremer 2009 [41]	Tomato soup	Soy sauce (Rich in glutamates)	17–33			
Ogasawara 2016 [42]	Mentsuyu (Japanese noodle soup)	Dried bonito (Rich in inosinate)	20			
Wang 2019 [25]	Chicken soup	MSG	20			
Leong 2015 [43]	Mee soto broth	MSG	22			
Jinap 2016 [44]	Spicy soup	MSG	32.5			
Carter 2011 [45]	Chicken broth	CDG	38			
Ball 2002 [46]	Pumpkin soup	CDG	40			
Yamaguchi 1984 [21]	Japanese clear soup	MSG	40			
Roininen 1996 [47]	Minestrone; leek and potato soup	MSG, inosinate, guanylate	40			
Rodrigues 2014 [48]	Garlic and salt	MSG	50	Spices and other	50	4.1
JPA 59–118038 [49]	Processed meat and fish	MSG, inosinate	30–40	Processed fish (including salted fish, canned fish, fish boiled in soy sauce and fish sausage)	30–50	3.5
de Quadros 2015 [50]	Fish burgers (minced fish)	MSG	50			
Wooward 2003 [51]	Sausage	CDG	17	Ham and sausage	17–75	0.3
JPA 59–118038 [49]	Processed meat and fish	MSG, inosinate	30–40			
dos Santos 2014 [52]	Sausages	MSG	75			
Miller 2014 [53]	Minced beef	Mushrooms (Rich in glutamates)	25	Beef	25	0.3
JPA60-153751 [54]	Pickled vegetable	MSG	55	Pickled vegetable	55	0.3
Rodrigues 2014 [55]	Mozzarella cheese	MSG	54	Cheese	54–100	0.4
da Silva 2014 [56]	Cream cheese	MSG	up to 100			
de Souza 2014 [57]	Butter	MSG	up to 100	Butter	100	0.0
Goncalves 2017 [58]	Margarine	MSG	33	Margarine	33	0.0
Kongsta 2020 [24]	Potato chips	MSG	30	Other confectionery	30–57	0.3
Buechler 2020 [23]	Chips and rice puffs	MSG, inosinate, guanylate	57			

*CDG* Calcium diglutamate, *JPA* Japanese Patent Application, *MSG* Monosodium glutamate, *NHNS* National Health and Nutrition Survey

### Statistical analysis

We first constructed a linear regression model with overall daily salt intake (g/day, continuous) as a dependent variable. To estimate the salt intake contribution from the 13 food groups (continued) to the overall salt intake, we included age (continuous), sex (dichotomous) and food intake (g/day) from the 13 food groups and the food items (continuous) in the following model (1).

1 $${Y}_{i}=\alpha +{\beta }_{1}{X}_{1i}+{\beta }_{2}{X}_{2i}+\cdots +{\beta }_{13}{X}_{13i}+{Z}_{i}\gamma +{\epsilon }_{i}$$

where ${Y}_{i}$ and ${X}_{ji}$ (for j = 1, …,13) are the overall daily salt intake and the food intake (g/day) from each of the 13 food groups for the *i*th individual, respectively. ${Z}_{i}$ is the covariate vector for the remaining 35 food items, sex and age for the *i*th individual. $\alpha , {\beta }_{j}, \gamma$ are regression coefficients and ${\epsilon }_{i}$ is a gaussian error term. The regression coefficients are estimated by ordinary least squared method.

The food group-specific upper and lower changes in salt intake by umami substance incorporation were estimated using the current market share of low-sodium products for the *j*th food group (denoted as ${M}_{j}$), the upper and lower salt intake reduction rates for the *j*th food group (denoted as ${U}_{j}$ and ${L}_{j}$, respectively), as well as the scenario-based increased consumption (denoted as ${S}_{k}$= 30, 60 or 100% increase (*k* = 1, 2, 3)), as follows.

$$Upper\ changes\ in\ salt\ intake\ of\ the\ jth\ food\ group\ under\ the\ kth\ scenario$$

$$={\widehat{\beta }}_{j}-{\widehat{\beta }}_{j}\times {U}_{j}\times \left({S}_{k}-{M}_{j}\right),$$

$$Lower\ changes\ in\ salt\ intake\ of\ the\ jth\ food\ group\ under\ the\ kth\ scenario$$

$$={\widehat{\beta }}_{j}-\widehat{\beta }_{j}\times {L}_{j}\times \left({S}_{k}-{M}_{j}\right),$$

The first term indicates the original salt intake contribution of the *j*th food group to the overall salt intake. In the second term, ${U}_{j}$ indicates how much salt intake we can reduce by incorporating umami substances into food groups. Finally, $\left({S}_{k}-{M}_{j}\right)$ indicates how much salt intake we can change into that from low-sodium products.

The baseline and reduced salt intakes among consumers of each food group, and the proportion of salt intake from each food group to the total salt intake were estimated for the total population, men, and women in the three scenarios.

The achieving rate of the salt intake reduction goals when umami substances are universally incorporated into all the food groups were then calculated by age groups and sex. Health Japan 21’s dietary goal is defined as a daily mean salt intake of less than 8 g [14], while that of the DRIs is 7.5 g for men and 6.5 g for women [15]. The WHO, on the other hand, recommends a daily salt intake of 5.0 g [59]. We used STATA version 16 for all analyses (Stata Corp LLC).

## Results

A total of 30,820 people joined the NHNS survey in 2016. We excluded ineligible subjects who were younger than 20 years old (*n* = 4,595) and consumed less than 1.5 g of salt per day (*n* = 46). 4,374 subjects had missing values on dietary intake. Finally, a sample of 21,805 Japanese persons with an average age of 57.8 (standard deviation [SD] 17.6) years were used in our analysis. Overall, the daily mean salt intake among the Japanese population was 9.95 g, which is higher than the daily salt intake recommended by Health Japan 21, the DRIs or the WHO.

The sex- and age-specific daily mean salt intake and the achieving rate of the salt intake reduction goals are shown in Table 2. Salt intake was likely to be higher among older persons than younger persons. Of the total population, 28.7% has already achieved the dietary goal of Health Japan 21, while 15.3% and 2.8% have achieved the dietary goals of the DRIs and the WHO, respectively. Men had higher salt intake than women in all age groups. The daily mean salt intake was the highest among men aged 60–69 years (11.43 g) and women aged 70–79 years (9.72 g), while the lowest among men (10.37 g) and women (8.60 g) both aged 20–29 years. The difference in daily mean salt intake between the highest and the lowest groups was 2.83 g. The rate of achieving the dietary goals was higher in women than men across all age groups.

**Table 2:** Daily mean salt intake among the NHNS participants and achieving rates of the salt reduction goals in Health Japan 21, the DRIs and the WHO, by age group and sex, the NHNS 2016

Age (years)	Number of the NHNS participants	Daily mean salt intake (g/day (SD))	Achieving rate of Health Japan 21 goal (%)^a^	Achieving rate of DRIs goal (%)^b^	Achieving rate of WHO goal (%)^c^
Total population					
20–29	1,480	9.44 (3.2)	35.3	19.3	3.9
30–39	2,548	9.52 (3.2)	35.0	20.6	4.1
40–49	3,394	9.49 (3.1)	33.8	19.6	3.9
50–59	3,254	9.86 (3.1)	29.0	14.0	2.5
60–69	4,940	10.52 (3.2)	21.5	10.0	1.5
70–79	3,934	10.38 (3.3)	23.8	12.5	2.1
80 +	2,255	9.58 (3.1)	33.5	18.6	3.5
All	21,805	9.95 (3.2)	28.7	15.3	2.8
Men					
20–29	707	10.37 (3.5)	24.2	19.0	2.1
30–39	1,205	10.51 (3.4)	23.7	18.0	1.8
40–49	1,581	10.47 (3.2)	23.3	17.5	1.8
50–59	1,481	10.77 (3.3)	18.7	13.2	1.6
60–69	2,304	11.43 (3.4)	13.3	9.0	0.4
70–79	1,802	11.09 (3.4)	17.5	13.3	1.2
80 +	892	10.32 (3.2)	24.2	19.3	2.1
All	9,972	10.83 (3.4)	19.4	14.4	1.4
Women					
20–29	773	8.60 (2.6)	45.4	19.5	5.4
30–39	1,343	8.64 (2.7)	45.1	22.9	6.1
40–49	1,813	8.65 (2.6)	43.0	21.5	5.8
50–59	1,773	9.09 (2.6)	37.6	14.7	3.2
60–69	2,636	9.72 (2.9)	28.6	11.0	2.4
70–79	2,132	9.78 (3.0)	29.2	11.8	2.8
80 +	1,363	9.10 (3.0)	39.6	18.2	4.4
All	11,833	9.21 (2.8)	36.5	16.0	4.0

*DRIs* Dietary Reference Intakes, *WHO* World Health Organization, *NHNS* National Health and Nutrition Survey, *SD* Standard deviation

^a^Recommend consumption of no more than 8 g of salt intake a day

^b^Recommend consumption of no more than 7.5 g of salt intake a day for men and 6.5 g for women

^c^Recommend consumption of no more than 5 g of salt intake a day

The sex-specific salt intake and potential reduction of salt intake estimated for each food group in three scenarios are presented in Table 3. The consumption of food items in each food group by the participants on any given day during the survey varied. The percentages of the participants who consumed the food items in each food group, i.e., “consumers,” were high for other seasonings (97.9%), soy sauce (85.9%), seasoning salt (83.1%), and miso paste (69.3%), and low for beef (24.71%), cheese (17.3%), butter (14.9%), margarine (14.4%) and other confectionery (15.2%). Compared to women, men were more likely to consume salt, soy sauce, spices and other, ham and sausage, beef, and pickled vegetable, and less likely to consume cheese and other confectionery. The consumers of soy sauce, seasoning salt, and miso paste took more than one gram of salt daily from each food group, and all participants consumed food items from at least one food group.

**Table 3:** Estimated mean salt intake from the 13 food groups and potential percentage reduction of salt intake by incorporation of umami substances into food items in the 30-, 60-, and 100-percent scenarios by sex, the NHNS 2016

	Number of the NHNS respondents (%)	Estimated mean salt intake from food group (g/day (SD)) ^a^	Potential percentage reduction of salt intake in scenarios					
			30-percent scenario		60-percent scenario		100-percent scenario	
			g/day	%^b^	g/day	%^b^	g/day	%^b^
Seasoning salt								
Total population	18,123 (83.1)	1.13 (1.00)	0.05–0.10	4.6–8.9	0.13–0.25	11.2–21.8	0.23–0.44	20.0–39.0
Men	8,482 (85.1)	1.26 (1.10)	0.06–0.11		0.14–0.27		0.25–0.49	
Women	9,641 (81.5)	1.02 (0.90)	0.05–0.09		0.11–0.22		0.20–0.40	
Soy sauce								
Total population	18,726 (85.9)	1.57 (1.30)	0.01–0.01	0.5–0.8	0.20–0.30	12.5–19.1	0.45–0.68	28.5–43.5
Men	8,736 (87.6)	1.71 (1.40)	0.01–0.01		0.21–0.33		0.49–0.74	
Women	9,990 (84.4)	1.44 (1.20)	0.01–0.01		0.18–0.28		0.41–0.63	
Miso paste								
Total population	15,105 (69.3)	1.48 (1.00)	0.04–0.09	2.6–6.2	0.11–0.25	7.1–16.7	0.19–0.45	13.1–30.7
Men	6,971 (69.9)	1.55 (1.00)	0.04–0.10		0.11–0.26		0.20–0.47	
Women	8,134 (68.7)	1.42 (0.90)	0.04–0.09		0.10–0.24		0.19–0.44	
Other seasoning								
Total population	21,341 (97.9)	0.34 (0.50)	0.01–0.04	2.9–10.4	0.02–0.08	6.2–22.4	0.04–0.13	10.6–38.4
Men	9,756 (97.8)	0.38 (0.50)	0.01–0.04		0.02–0.09		0.04–0.15	
Women	11,585 (97.9)	0.31 (0.40)	0.01–0.03		0.02–0.07		0.03–0.12	
Spices and other								
Total	5,567 (25.5)	0.08 (0.20)	0.01	13.0	0.02	28.0	0.04	48.0
Men	2,707 (27.1)	0.08 (0.20)	0.01		0.02		0.04	
Women	2,860 (24.2)	0.07 (0.10)	0.01		0.02		0.03	
Processed fish								
Total population	13,783 (63.2)	0.80 (0.80)	0.06–0.11	0.5–0.8	0.14–0.23	12.5–19.1	0.23–0.39	28.5–43.5
Men	6,393 (64.1)	0.86 (0.90)	0.07–0.11		0.15–0.24		0.25–0.42	
Women	7,390 (62.5)	0.75 (0.80)	0.06–0.10		0.13–0.21		0.22–0.36	
Ham and sausage								
Total population	8,899 (40.8)	0.66 (0.50)	0.03–0.15	8.0–13.3	0.07–0.29	17.0–28.3	0.11–0.49	29.0–48.3
Men	4,245 (42.6)	0.72 (0.60)	0.04–0.16		0.07–0.32		0.12–0.54	
Women	4,654 (39.3)	0.60 (0.50)	0.03–0.13		0.06–0.27		0.10–0.45	
Beef								
Total population	5,380 (24.7)	0.19 (0.20)	0.01	7.4	0.03	14.9	0.05	24.9
Men	2,717 (27.2)	0.21 (0.20)	0.02		0.03		0.05	
Women	2,663 (22.5)	0.17 (0.10)	0.01		0.03		0.04	
Pickled vegetable								
Total population	8,813 (40.4)	0.91 (1.00)	0.15	16.3	0.30	32.8	0.50	54.8
Men	4,390 (44.0)	0.94 (1.10)	0.15		0.31		0.51	
Women	4,423 (37.4)	0.89 (0.90)	0.14		0.29		0.49	
Cheese								
Total population	3,767 (17.3)	0.51 (0.40)	0.08–0.15	16.0–29.6	0.17–0.31	32.2–59.6	0.28–0.51	53.8–99.6
Men	1,551 (15.6)	0.52 (0.40)	0.08–0.15		0.17–0.31		0.28–0.51	
Women	2,216 (18.7)	0.51 (0.40)	0.08–0.15		0.16–0.31		0.28–0.51	
Butter								
Total population	3,253 (14.9)	0.24 (0.20)	0.07	30.0	0.14	60.0	0.24	100.0
Men	1,397 (14.0)	0.24 (0.20)	0.07		0.14		0.24	
Women	1,856 (15.7)	0.24 (0.20)	0.07		0.14		0.24	
Margarine								
Total population	3,144 (14.4)	0.45 (0.40)	0.04	9.9	0.09	19.8	0.15	33.0
Men	1,320 (13.2)	0.49 (0.40)	0.05		0.10		0.16	
Women	1,824 (15.4)	0.42 (0.30)	0.04		0.08		0.14	
Other confectionery								
Total population	3,323 (15.2)	0.10 (0.10)	0.01–0.02	8.9–16.9	0.02–0.04	17.9–34.0	0.03–0.06	29.9–56.8
Men	1,161 (11.6)	0.12 (0.10)	0.01–0.02		0.02–0.04		0.04–0.07	
Women	2,162 (18.3)	0.10 (0.10)	0.01–0.02		0.02–0.03		0.03–0.06	
All foods								
Total population	21,801 (100.0)	5.06 (2.70)	0.24–0.43	2.3–4.1	0.68–1.20	6.4–11.4	1.27–2.22	12.0–21.1
Men	9,970 (100.0)	5.57 (2.90)	0.26–0.47	2.3–4.2	0.74–1.31	6.5–11.5	1.39–2.43	12.1–21.3
Women	11,831 (100.0)	4.63 (2.40)	0.22–0.40	2.3–4.1	0.63–1.10	6.4–11.3	1.16–2.03	11.9–20.9

*SD* Standard deviation

^a^Salt intake from each food group among consumer, not all participants

^b^The reduction rate of each food group was the same for men and women. The salt reduction rate for the total population was the average reduction rate among individuals in the percentage decrease of overall salt intake

In the universal incorporation scenario where consumers could potentially reduce their salt intake up to 100% with the additional umami substances, the highest amount of expected salt reduction was found in soy sauce (0.45–0.68 g), followed by cheese (0.28–0.51 g), pickled vegetable (0.50 g), ham and sausage (0.11–0.49 g), seasoning salt (0.23–0.44 g) and miso paste (0.19–0.45 g). Negligible reductions in salt intake could be expected for spice and others, beef, and other confectionery (< 0.1 g).

Table 4 presents proportion of salt intake from each food group to overall salt intake, and potential salt intake reduction with the additional umami substances in the universal incorporation scenario by sex. Among all participants, soy sauce (12.5%), miso paste (9.7%), and seasoning salt (8.9%) were the major contributors to the overall daily salt intake. In contrast, spice and others, beef, cheese, butter, margarine, and other confectionery were the minor sources of salt intake (< 1%). Although high reduction of salt intake was found in cheese among the cheese consumers (0.28–0.51 g), there was less impact at the population level (0.05–0.09 g) because there were few cheese consumers among the participants. The total daily mean salt intake from all the food groups was 5.06 g for all the participants, resulting in a 48.0% salt intake contribution to the overall salt intake. Thus, by universally incorporating umami substances into the food groups, salt intake could be reduced by an average of 1.27–2.22 g among the total population (with an average reduction rate of 12.0–21.1%). This corresponds to a reduction of 12.8–22.3% in the average salt intake among the total population (not shown in the table).

**Table 4:** Proportion of salt intake from each food group contributed to overall salt intake, and potential percentage reduction of salt intake with the additional umami substances in the universal incorporation scenario by sex, the NHNS 2016

	All (*n* = 21,805)		
	Proportion of salt intake contributed to overall salt intake (%)	Reduction in salt intake^a^	
		g/ day	%
Seasoning salt			
Total population	8.9	0.19–0.37	1.8–3.5
Men	9.4	0.21–0.42	1.9–3.7
Women	8.6	0.17–0.32	1.7–3.4
Soy sauce			
Total population	12.5	0.38–0.59	3.6–5.4
Men	12.2	0.43–0.65	3.7–5.6
Women	12.8	0.35–0.53	3.5–5.3
Miso paste			
Total population	9.7	0.13–0.31	1.3–3.0
Men	9.9	0.14–0.33	1.3–2.9
Women	9.5	0.13–0.30	1.3–3.0
Other seasoning			
Total population	3.2	0.04–0.13	0.3–1.2
Men	3.2	0.04–0.14	0.3–1.3
Women	3.3	0.03–0.12	0.3–1.2
Spices and other			
Total population	0.2	0.01	0.1
Men	0.2	0.01	0.1
Women	0.2	0.01	0.1
Processed fish			
Total population	4.8	0.15–0.24	1.4–2.3
Men	4.8	0.16–0.27	1.4–2.3
Women	4.9	0.14–0.23	1.4–2.3
Ham and sausage			
Total population	2.6	0.05–0.20	0.5–2.0
Men	2.5	0.05–0.23	0.5–2.1
Women	2.8	0.04–0.18	0.4–1.9
Beef			
Total population	0.5	0.01	0.1
Men	0.4	0.01	0.1
Women	0.6	0.01	0.1
Pickled vegetable			
Total population	3.3	0.20	1.8
Men	3.2	0.23	1.9
Women	3.4	0.18	1.8
Cheese			
Total population	0.9	0.05–0.09	0.5–0.9
Men	1.1	0.04–0.08	0.4–0.8
Women	0.8	0.05–0.10	0.6–1.1
Butter			
Total population	0.4	0.04	0.4
Men	0.4	0.03	0.3
Women	0.3	0.04	0.4
Margarine			
Total population	0.7	0.02	0.2
Men	0.8	0.02	0.2
Women	0.6	0.02	0.3
Other confectionery			
Total population	0.2	0.00–0.01	0.0–0.1
Men	0.2	0.00–0.01	0.0–0.1
Women	0.1	0.01–0.01	0.0–0.1
All foods			
Total population	48.0	1.27–2.22	12.0–21.1
Men	47.5	1.39–2.43	12.1–21.3
Women	48.6	1.16–2.03	11.9–20.9

^a^The scenario in which umami substances were universally incorporated into selected food groups. The salt reduction rate was the average among individuals

**Fig. 1:**
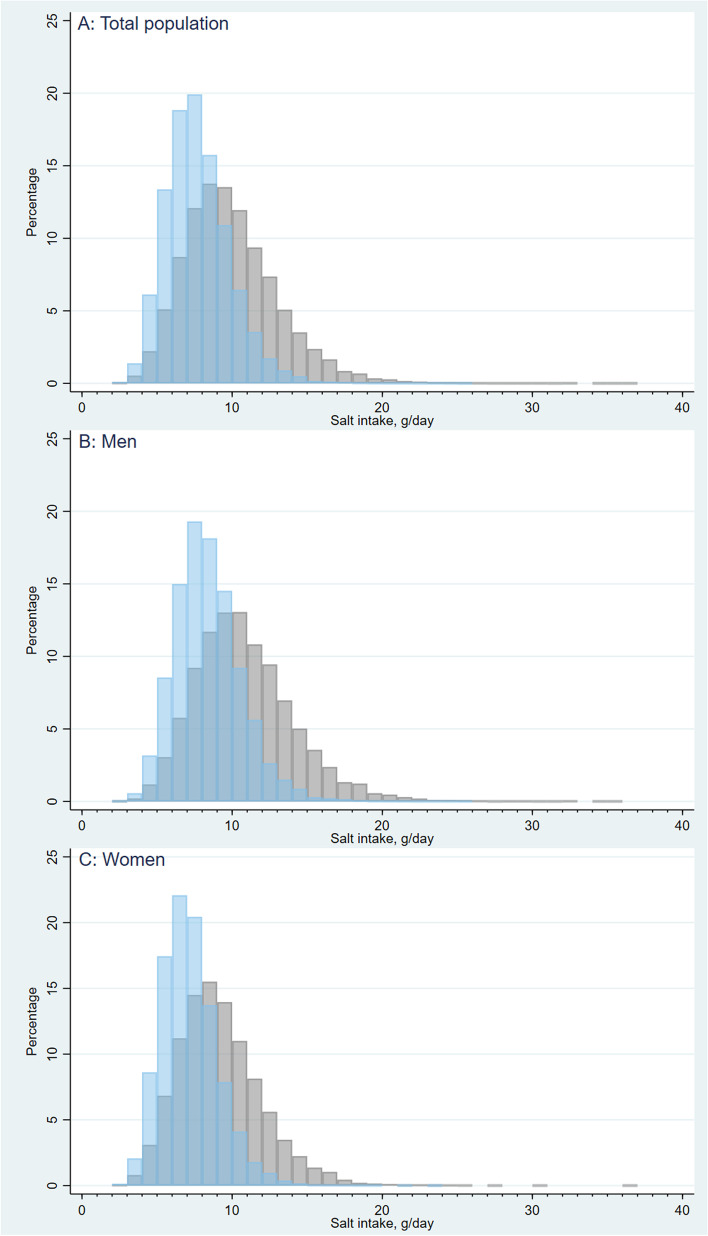
Distributions of daily salt intake among the total and sex-specific population before and after the universal incorporation of umami substances into food items in (**A**) the total population, by sex of (**B**) men, and (**C**) women, NHNS 2016. The grey bars indicate the distributions of daily salt intake before the universal incorporation of umami substances into food items. The blue bars indicate the distributions of daily salt intake after the universal incorporation of umami substances into food items

Figure 1 describes distributions of daily salt intake among the total and sex-specific population before and after the universal incorporation of umami substances into food items. It is obvious that higher proportions of the population in both sexes came to consume less amount of salt intake after the universal incorporation of umami substances into food groups. Table 5 shows sex- and age-specific daily mean salt intake estimated after umami substances are universally incorporated into the food items and their achieving rates of the salt reduction goals set by Health Japan 21, the DRIs and the WHO. While the salt intake still varied by sex and age groups, the difference in the mean salt intake between the highest and the lowest groups was slightly smaller when umami substances were universally incorporated into food items. The daily mean salt intake of all the participants in the universal incorporation scenario was 7.73–8.68 g; thereby, suggesting a possibility to achieve Health Japan 21’s goal of consuming less than 8 g of daily salt intake by 2023. Moreover, the rate of those who achieve Health Japan 21’s dietary goal increased from 19.6% to 31.2–46.6% for men and 36.9% to 53.6–70.8% for women under the scenario. While approximately 23.9–36.7% of men and 25.6–39.2% of women could achieve the recommended daily salt intake outlined by the DRIs, only 2.2–3.8% of men and 6.4–10.8% of women were expected to achieve the WHO’s dietary goal: 5 g of daily salt intake, in the universal incorporation scenario.

**Table 5:** Estimated salt intake and achieving rates of the dietary goals with the additional umami substances in the universal incorporation scenario by sex and age groups, the NHNS 2016

Age (years)	Estimated salt intake (g/day)	Achieving rate of Health Japan 21 goal (%)^a^	Achieving rate of DRIs goal (%)^b^	Achieving rate of WHO goal (%)^c^
Total population				
20–29	7.40–8.34	49.5–68.0	31.1–45.7	6.1–10.0
30–39	7.46–8.41	48.9–63.9	30.3–44.3	5.9–10.6
40–49	7.42–8.36	49.4–65.0	29.6–44.1	6.1–10.1
50–59	7.69–8.64	44.3–60.9	24.1–38.6	3.9–7.0
60–69	8.13–9.13	35.1–52.4	18.1–29.7	2.7–4.8
70–79	8.02–8.98	38.5–54.7	20.9–33.1	3.4–5.4
80 +	7.43–8.29	49.2–65.0	29.8–43.2	5.7–9.6
All	7.73–8.68	43.4–59.7	24.8–38.1	4.4–7.6
Men				
20–29	8.12–9.15	36.9–54.2	29.8–44.4	3.1–5.1
30–39	8.19–9.25	35.2–50.3	27.7–39.9	2.4–5.0
40–49	8.15–9.20	35.2–50.2	27.1–41.4	3.0–4.8
50–59	8.38–9.43	31.1–46.9	22.6–36.5	2.6–3.9
60–69	8.79–9.90	24.3–39.2	17.7–29.1	1.0–2.1
70–79	8.58–9.61	28.9–44.0	21.8–34.4	1.8–2.9
80 +	8.00–8.92	37.3–53.3	30.8–42.8	3.0–5.2
All	8.40–9.45	31.2–46.6	23.9–36.7	2.2–3.8
Women				
20–29	6.75–7.60	61.1–80.6	32.3–47.0	8.9–14.5
30–39	6.80–7.64	61.2–76.0	32.5–48.2	9.0–15.7
40–49	6.79–7.62	61.8–77.9	31.7–46.4	8.8–14.7
50–59	7.11–7.97	55.3–72.6	25.4–40.4	5.0–9.6
60–69	7.54–8.45	44.5–63.8	18.5–30.2	4.1–7.2
70–79	7.54–8.45	46.6–63.8	20.2–32.0	4.8–7.6
80 +	7.06–7.88	57.0–72.7	29.1–43.4	7.5–12.5
All	7.17–8.04	53.6–70.8	25.6–39.2	6.4–10.8

*SD* Standard deviation, *DRIs* Dietary Reference Intakes, *WHO* World Health Organization

^a^Recommend consumption of no more than 8 g of salt intake a day

^b^Recommend consumption of no more than 7.5 g of salt intake a day for men and 6.5 g for women

^c^Recommend consumption of no more than 5 g of salt intake a day

## Discussion

Excess salt intake reduction is now a global public health challenge [60]. Reducing salt intake has been identified as one of the most cost-effective measures to improve population health outcomes [59]. High sodium intake is a crucial risk factor for chronic diseases, and it has posed a high burden in Japan for decades [8, 10]. The current daily mean salt intake in Japan exceeds public recommendations across all ages and in both sexes. This study shows that it is possible to reduce the Japanese population’s salt intake by up to 2.22 g (21.1%) on average without compromising the taste of food by substituting salt with umami substances, which corresponds to a 22.3% reduction in the average salt intake of the population. In addition to reducing the salt intake among consumers, this study demonstrates that the universal incorporation of umami substances into the some foods can effectively reduce salt intake at the population level.

The previous study, using the data from the National Health and Nutrition Examination Survey 2013–2016 in the US, focused solely on MSG as a solution for salt reduction [26]. However, global recognition of MSG as an effective and practical solution for salt intake reduction remains a major challenge. In a widely reported study published in 1968, MSG in Chinese food was suggested to be the cause behind numbness and palpitations in the neck and arms and linked to various health problems, known as the Chinese restaurant syndrome [61]. Following this study, several studies also reported the association between MSG and various health effects, including asthma, urticaria, atopic dermatitis, dyspnea, tachycardia, metabolic syndrome, obesity and blood pressure increase [62–66]. However, other studies, including a double-blind placebo-controlled trial, have evaluated the reported reactions to MSG and confirmed a lack of plausible evidence between MSG intake and the development of such symptoms [67–70]. Furthermore, major scientific committees and regulatory bodies, such as the Joint FAO/WHO Expert Committee on Food Additives (JECFA), the European Commission Scientific Committee on Food (SCF), and the U.S. Food and Drug Administration (FDA), have assessed the safety of MSG and all separately came to a conclusion that MSG is safe to consume at a normal intake level and there is no evidence linking the use of MSG to long-term medical problems for the general public [71]. The more recent evidence-based safety reviews of MSG also came to the same conclusions, addressing that some studies speculatively linked animal pharmacology to human food use of MSG, and many are based on excessive dosing that does not meet with levels normally consumed in food products [72, 73].

The previous US study focusing solely on MSG reported that the overall salt intake reduction among the population was 7.3% [26]. Meanwhile, reduction of sodium can also be achieved with sodium-free glutamates, such as CDG, inosinate, and guanylate [74]. Accordingly, the scope of our study has been expanded from MSG to the wider range of umami substances. As such, our findings suggested that umami substances could potentially make a greater impact on reducing salt intake than MSG in the previous study. On the other hand, we selected food items that are widely consumed by the Japanese, such as seasoning salt, soy sauce, miso paste, other seasonings, and processed fish. Indeed, soy sauce is one of the most highly consumed food items in Japan, and this study showed the largest impact of daily salt reduction in soy sauce, up to 0.68 g among its consumers and 0.37 g among the total population. On the other hand, cheese, spices and other, beef, margarine, and other confectionery had less impact on reducing salt intake at the population level because they are less consumed in Japan.

To reduce the Japanese population’s daily salt intake, the Japanese government took steps to enforce the new food labelling system and the nutrition labelling system in April 2020 [75, 76]. These systems made it mandatory for food companies to disclose the amount of salt/sodium in their products to ensure that their consumers are aware of the nutritional contents in their foods. However, these measures alone may not be sufficient in addressing the problem because reducing salt intake is not a priority among consumers [77]. Furthermore, reducing the salt in foods may lower the quality of food. For example, a 75% reduction of salt in sausages decreases the sausages' hardness, chewiness, and cohesiveness [52]. Hence, food companies often provide low-sodium alternatives that give their consumers the taste and the quality they seek without the harmful amounts of sodium [78]. Potassium chloride, calcium chloride, and magnesium sulfate are also commonly used as substitutes for table salt. However, their bitter taste has repelled the consumers and resulted in their limited use. In contrast, umami substances, which are naturally present in various foods, are widely accepted by consumers [79]. As umami substances enhance the original flavor in foods, incorporation of umami substances into food items will reduce the salt intake more effectively [18, 80].

The food industry should take action to raise consumer awareness on the benefits of eating low-sodium foods while reducing the salt in their products, so that consumers can adapt to the changes in the taste over time [74]. Accordingly, the food industry's role is essential in reducing the daily salt intake of Japanese population and reducing their health risks [11]. Moreover, reducing salt intake through food science and technological advance is an appropriate method to make the most impactful salt intake reduction at the population level [60]. Our study has provided the essential data on the distribution of the selected food consumers, the market shares of the selected food items with low-sodium alternatives, and its impact on public health by showing the potential salt intake reduction. This information may instruct and inspire the food industry to develop more low-sodium products and distribute them in the market.

This study has some strengths. This is the first study to show the impact of salt reduction by replacing NaCl with umami substances in the selected Japanese food products. The use of the nationally representative data has guaranteed the study's generalisability to the Japanese population. The modelling assumptions of salt reduction were made based on scientific evidence, consultation with food scientists and consideration of market distributions of low-sodium products.

This study is subject to similar limitations found in other studies concerning dietary patterns [81, 82]. First, the dietary data we used in our analysis may have some biases. Because the dietary data from the NHNS were based on the weighted single-day dietary record, the analysis may not have captured the long-term dietary patterns. In dietary surveys, participants' self-reports tend to be associated with social desirability and recall bias. Moreover, reliance on dietary intake records made by household representatives may lead to biased estimates of dietary intake, particularly for those meals taken outside the home. Additionally, NHNS's stratified two-cluster sampling design may have caused selection bias, leading to biased estimates. Second, the data on food-specific salt intake were not publicly available. Therefore, an individual's food-specific salt intake was estimated by regression method which may not have accurately reflected the actual amount of salt intake from each food item. Third, age-specific preferences of food which may affect the potential overall salt reduction were not considered [83]. Fourth, we did not consider possible changes in food intake as a result of umami substance incorporation to reduce salt intake. Umami flavour may increase overall food intake or decrease vegetable intake as the previous studies suggested that vegetable intake is associated with salt intake [84–86]. The changes in food intake could change the effects of umami substances on salt intake reduction. Fifth, we did not include the low-sodium products using sodium replacers, other than umami substances, such as potassium chloride, mineral salts and yeast extracts in Japan [87–89] in our modelling, as we assessed the effects of umami substances on salt intake reduction. Thus, we may have underestimated the market share of low-sodium products. Finally, we did not consider the Japanese population’s current MSG intake and its association with health outcomes due to unavailability of data. Hence, caution is needed when the salt intake reduction is pursued by using MSG which may cause the long-term health effects on the population.

## Conclusions

Our study has suggested that the incorporation of umami substances into the selected food items could potentially reduce the average daily salt intake of the Japanese population by 22.3%, which is equivalent to 2.22 g of daily salt reduction. The universal incorporation of umami substances into the selected food items might enable the Japanese to achieve the national dietary goals. However, the level of salt intake reduction still falls short of 5.0 g/day recommended by WHO. Along with the public health efforts, collaboration with experts in food science should be pursued. Further investigation, innovation, and distribution of low-sodium food products are needed to help reduce the adult Japanese population's salt intake and consequently reduce their chances of developing NCDs.

## Data Availability

The data that support the findings of this study are available from MHLW, but restrictions apply to the availability of these data. The data were used with an approval from MHLW to be utilized for the current study without making it publicly available. Further information on application and use of data can be obtained by contacting the corresponding author.

## Abbreviations

CDG: Calcium diglutamate
CVDs: Cardiovascular diseases
DRIs: Dietary Reference Intakes
FDA: Food and Drug Administration
GBD: Global Burden of Disease
JECFA: Joint FAO/WHO Expert Committee on Food Additives
MHLW: Ministry of Health, Labour and Welfare
MSG: Monosodium glutamates
NaCl: Sodium chloride
NCDs: Non-communicable diseases
NHNS: National Health and Nutrition Survey
NNS: National Nutrition Survey
SCF: European Commission Scientific Committee on Food
SD: Standard deviation
US: Unites States
WHO: World Health Organization
